# Reply to Perrykkad and Hohwy: When big data are the answer

**DOI:** 10.1073/pnas.1903773116

**Published:** 2019-07-03

**Authors:** David M. Greenberg, Varun Warrier, Carrie Allison, Simon Baron-Cohen

**Affiliations:** ^a^Autism Research Centre, Department of Psychiatry, University of Cambridge, Cambridge CB2 8AH, United Kingdom

Perrykkad and Hohwy (1) argue that autism was historically diagnosed predominantly in males (4 males to 1 female) (2) and thus the defining characteristics of autism are male biased. They conclude that because the Autism Spectrum Quotient (AQ) was developed within this historical framework, (*i*) the AQ may not capture the phenotypic profile of autistic females and (*ii*) the AQ has a male bias. We agree that autism has been historically underdiagnosed in females but disagree with their conclusions for at least 5 reasons.

First, our study (3) shows that regardless of sex, autistic individuals have, on average, high AQ scores. Multiple studies (4, 5) show that autistic females score as high or higher on the AQ than autistic males. Second, items on the AQ fall into 5 subscales, none of which has a built-in male bias. Third, latent trait analysis (6) suggests that a short version of the AQ detects autistic traits equivalently in autistic males and females. Fourth, sex differences in autistic individuals may reflect biological differences. For example, autistic traits are positively correlated with levels of prenatal testosterone, even within one sex (7). On average, prenatal testosterone is produced at higher levels in males and is elevated in fetuses who go on to be diagnosed with autism (8). Further, autistic females, on average, have a higher burden of de novo protein-truncating variants (9) and copy number variants (10), supporting the female protective effect hypothesis (11). Fifth, the AQ was developed to measure autistic traits in the general population, and not typical sex differences. In summary, to conclude that the AQ will have an inherent male bias in the typical population is incorrect. An alternative hypothesis is that there is a real sex difference in the average number of autistic traits in typical males and females. Consistent with this latter hypothesis is that typical males, on average, score higher on a number of instruments that measure autistic traits that were developed at very different time points and with very different item content.

Perrykkad and Hohwy (1) further suggest that the 3 additional measures we used—the 10-item short forms of the Empathy Quotient (EQ-10), Systemizing Quotient-Revised (SQ-R-10), and Sensory Perception Quotient (SPQ-10)—were all developed “with reference to their expected relationship with the AQ.” However, these measures were not developed to have an expected relationship with the AQ. Further, Perrykkad and Hohwy overlook that we developed the SQ-R-10 based on a 44-item gender-neutral version of the SQ-R. We did this because we are aware of the risk of gender-stereotype bias influencing the design of measures, yet it still showed a sex difference. Perrykkad and Hohwy also neglect to consider that we replicated our findings in a validation cohort using different versions of the EQ and SQ. This suggests that the results are to some extent independent of which items are included and rather an indication of effects in the underlying domains.

We are grateful to Perrykkad and Hohwy (1) for stimulating interesting discussion on how to interpret the findings from our big data of over 600,000 typical individuals and from 36,000 autistic people.
